# Statin use and breast cancer survival and risk: a systematic review and meta-analysis

**DOI:** 10.18632/oncotarget.5557

**Published:** 2015-10-12

**Authors:** Qi-Jun Wu, Chao Tu, Yuan-Yuan Li, Jingjing Zhu, Ke-Qing Qian, Wen-Jing Li, Lang Wu

**Affiliations:** ^1^ Department of Clinical Epidemiology, Shengjing Hospital of China Medical University, Shenyang, Liaoning 110004, China; ^2^ Oncology Institute, the Affiliated Hospital of Nanjing Medical University, Changzhou No.2 People's Hospital, Changzhou, Jiangsu 213003, China; ^3^ Department of Hematology, the Affiliated Hospital of Xuzhou Medical College, Xuzhou, Jiangsu 221000, China; ^4^ Program of Quantitative Methods in Education, University of Minnesota, Minneapolis, Minnesota 55455, USA; ^5^ Division of Epidemiology, Department of Medicine, Vanderbilt Epidemiology Center, Vanderbilt-Ingram Cancer Center, Vanderbilt University School of Medicine, Nashville, Tennessee 37203, USA; ^6^ Center for Clinical and Translational Science, Mayo Clinic, Rochester, Minnesota 55905, USA

**Keywords:** statin, breast cancer, risk, mortality, meta-analysis

## Abstract

The purpose of this study is to determine the associations between statin use and breast cancer survival and risk by performing a systematic review and meta-analysis. We searched PubMed, Embase and Web of Science up to August 2015 for identifying relevant prospective or case-control studies, or randomized clinical trials. Five prospective studies involving 60,911 patients reported the association between statin use and breast cancer mortality. Eleven prospective studies, 12 case-control studies and 9 randomized clinical trials involving 83,919 patients reported the association between statin use and breast cancer risk. After pooling estimates from all available studies, there was a significantly negative association between pre-diagnosis statin use and breast cancer mortality (for overall survival (OS): hazard ratio (HR) = 0.68, 95% confidence interval (CI) 0.54–0.84; for disease specific survival (DSS): HR = 0.72, 95% CI 0.53–0.99). There was also a significant inverse association between post-diagnosis statin use and breast cancer DSS (HR = 0.65, 95% CI 0.43–0.98), although the association with breast cancer OS did not reach statistical significance (HR = 0.71, 95% CI 0.48–1.07). Additionally, there was a non-linear relationship for the duration of post-diagnosis statin use with breast cancer specific mortality. On the other hand, with regards to the relationship between statin use and breast cancer risk, no significant association was detected. Our analyses suggest that although statin use may not influence breast cancer risk, the use of statin may be associated with decrease mortality of breast cancer patients. Further large-scale studies are warranted to validate our findings.

## INTRODUCTION

Breast cancer remains the most common and death-causing cancer in females [1]. In US it is expected that there will be approximately 231,840 new cases and 40,290 deaths from breast cancer among females in 2015 [1]. It is critical to take appropriate actions for the chemoprevention and chemotherapy of this malignant cancer, at the aim of decreasing its public health burden. As a class of drugs for managing cardiovascular diseases, statins are widely used all over the world [2]. Preclinical studies have demonstrated that statins can potentially suppress tumor and reduce metastatic potential of breast cancer [3–6]. These seem to support the beneficial effect of statin use on survival of breast cancer patients. However, findings from epidemiological studies are not consistent. Two cohort studies suggest that both pre-diagnosis statin use and post-diagnosis statin use are associated with reduced risks of overall mortality and breast cancer-specific mortality [7, 8]. On the other hand, three other cohort studies do not suggest the beneficial effects of statin use on breast cancer patients' survival [9–11]. A meta-analysis summarizing available evidence will be critical for clarifying the relationship between statin use and survival outcome of breast cancer patients.

Similarly, with regards to the risk of breast cancer development, laboratory studies have suggested statins to be potentially chemopreventive [12, 13], while this is supported by only a proportion of relevant clinical studies. A meta-analysis of seven randomized clinical trials (RCTs) and nine observational studies does not identify such a relationship [14]. Another meta-analysis study summarizing 24 observational studies also suggests a null association [2]. Although in this meta-analysis a relatively large number of subjects is involved, several more recent studies evaluating the relationship between statin use and breast cancer risk have been published [15–17]. An updated meta-analysis summarizing all available evidence including those from recently published studies is thus necessary to more accurately clarify the relationship.

We aim to conduct a comprehensive meta-analysis study to assess the associations between statin use and breast cancer survival and risk. Findings from such a study may help determine whether statins could be used for either chemotherapy or chemoprevention of breast cancer, at the aim of reducing health burden from this malignant disease.

## RESULTS

### Literature search and study characteristics

**Figure 1:**
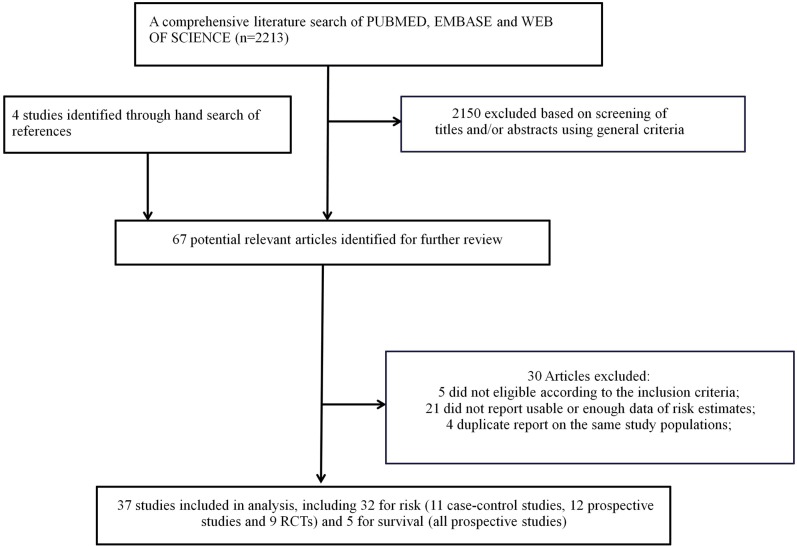
Flow chart for selection of eligible studies

The detailed steps of our literature search and article screening were shown in Figure 1. A total of 32 studies evaluating breast cancer risk [15–46] and 5 studies evaluating breast cancer survival [7–11] met the inclusion criteria and were included in the current meta-analysis. Several studies evaluated endpoints beyond the scope of our hypothesis like disease recurrence and thus were not included in the current meta-analysis [47–50]. Several studies reported the association estimates separately according to different subgroups and the combined effect sizes were not able to be determined based on available information [8, 17, 28]. We thus treated these estimates separately and incorporated all of them in the pooled analyses. The detailed characteristics of the included studies were shown in Tables 1 and 2 respectively. For breast cancer mortality, 5 prospective studies were available. Three were conducted in Europe and 2 in America. These studies enrolled 60,911 patients and had a median follow up of 5.3 years (range 2.9–11.5 years). All these studies were categorized as high quality studies, according to the Newcastle-Ottawa Quality Assessment Scale (NOS) (Supplementary Table 1). For breast cancer risk, a total of 11 case-control studies, 12 prospective studies, and 9 RCTs were available. Overall, 20 studies were conducted in America, 9 in Europe, 2 in Asia, and 1 in Oceania. The studies enrolled 83,919 patients and had a median follow up of 5 years (range 2.2–10.8 years). Among the observational studies, 11 of the 12 prospective studies (91.7%) and 9 of the 11 case-control studies (81.8%) were categorized as high quality studies (Supplementary Tables 2 and 3). However, only 2 of the 9 RCTs (22.2%) were categorized as studies with low risk of bias (Supplementary Table 4).

**Table 1:** Characteristics of studies evaluating statin use and breast cancer mortality

Author,publication year, location	Study type	Observed patients, follow up time	Categoriesof exposure/reference	HR (95% CI) for mortality	Matched/adjusted variables
Brewer, 2013, Texas, US	CS	723, median 2.9 yrs	No statin use after diagnosis Statin use after diagnosis OS DSS	1.0 (Ref) 1.00 (0.63, 1.60) 0.95 (0.58, 1.56)	lymphatic/vascular invasion for PFS and lymphatic/vascular invasion, nuclear grade and surgery within 1 year, radiation therapy, hormonal receptor status and HER2 status
Cardwell, 2014, UK	CS	17880, mean 5.7 yrs	No statin use after diagnosis Statin use after diagnosis DSS 1-365ddd 365+ ddd OS 1-365ddd 365+ ddd No statin use before diagnosis Statin use before diagnosis DSS 1-365ddd 365+ ddd OS 1-365ddd 365+ ddd	1.0 (Ref) 0.75 (0.65, 0.86) 0.75 (0.62, 0.90) 0.75 (0.64, 0.88) 0.78 (0.71, 0.86) 0.78 (0.69, 0.89) 0.79 (0.70, 0.88) 1.0 (Ref) 0.81 (0.71, 0.93) 0.80 (0.69, 0.94) 0.84 (0.68, 1.05) 0.78 (0.70, 0.86) 0.76 (0.68, 0.86) 0.81 (0.69, 0.95)	year of diagnosis, age at diagnosis, surgery within 6 months, radiotherapy within 6 months, chemotherapy within 6 months, hormone therapy use, comorbidities, and other medication usage
Murtola, 2014, Finland	CS	31236, median 3.25 yrs	No statin use after diagnosis Statin use after diagnosis DSS (for localized cases at diagnosis) 10–322 ddd 333–800 ddd 801+ ddd DSS (for metastatic cases at diagnosis) 10–322 ddd 333–800 ddd 801+ ddd OS (for localized cases at diagnosis) 10–322 ddd 333–800 ddd 801+ ddd OS (for metastatic cases at diagnosis) 10–322 ddd 333–800 ddd 801+ ddd No statin use before diagnosis Statin use before diagnosis DSS 1–495 ddd 496+ ddd OS (for localized cases at diagnosis) 1–495 ddd 496+ ddd OS (for metastatic cases at diagnosis) 1–495 ddd 496+ ddd	1.0 (Ref) 0.35 (0.28, 0.45) 0.54 (0.40, 0.72) 0.43 (0.31, 0.61) 0.42 (0.28, 0.62) 0.49 (0.33, 0.73) 0.66 (0.42, 1.01) 0.37 (0.18, 0.79) 0.24 (0.03, 1.74) 0.39 (0.33, 0.46) 0.56 (0.45, 0.69) 0.48 (0.38, 0.61) 0.37 (0.27, 0.50) 0.55 (0.39, 0.78) 0.73 (0.49, 1.08) 0.41 (0.21, 0.80) 0.38 (0.09, 1.53) 1.0 (Ref) 0.54 (0.44, 0.67) 0.69 (0.55, 0.86) 0.40 (0.29, 0.56) 0.58 (0.49, 0.70) 0.69 (0.56, 0.84) 0.51 (0.39, 0.68) 0.66 (0.47, 0.92) 0.91 (0.63, 1.31) 0.58 (0.37, 0.92)	age, tumor characteristics, and treatment selection
Nickels, 2013, German	CS	3189, median 5.3 yrs	No statin use after diagnosis Statin use after diagnosis OS DSS	1.0 (Ref) 1.21 (0.87, 1.69) 1.04 (0.67, 1.60)	Age, tumor size, nodal status, metastases, menopausal hormone treatment, mode of detection, radiotherapy, and smoking, cardiovascular disease, diabetes, BMI
Desai, 2015, US	CS	7883, 11.5 yrs	No statin use at baseline (before diagnosis) Statin use at baseline (before diagnosis)DSS	1.0 (Ref) 0.91 (0.60, 1.37)	Race, education, smoking, BMI, waist circumference, mammogram in the past 2 yrs, Gail 5-yr risk, female relative with breast cancer, age at menarche, number of live births, breast biopsy, hysterectomy, hormone use, oral contraceptive, aspirin use, study component

CI: confidence interval; CS: cohort study; Ref: reference; HR: hazard ratio; PFS: Progression free survival; OS: overall survival; DSS: disease specific survival; ddd: daily defined doses; HER2: human epidermal growth factor receptor-2; BMI, body mass index

**Table 2:** Characteristics of studies evaluating statin use and breast cancer risk

Author, publication year, location	Study type	Cases/subject or control (age), duration of follow up	Categoriesof exposure/reference	RR (95% CI)	Matched/adjusted variables
McDougall, 2013, Seattle–Puget Sound, US	PC-CS	IDC: 916/902 (55–74 y) ILC: 1068/902 (55–74 y)	IDC: Never use Ever use (for ≥0.5 y) ILC: Never use Ever use (for ≥0.5 y)	1.0 (Ref) 1.16 (0.92–1.47) 1.0 (Ref) 1.22 (0.98–1.53)	Age, county of residence, reference year, HRT
Graaf, 2004, Netherlands	PC-CS	467/16976 (NA)	No statin use statin use	1.0 (Ref) 1.07 (0.65–1.74)	Sex, year of birth, geographic region, duration of follow-up, index date, diabetes mellitus, prior hospitalizations, chronic disease score, chronic use of diuretics, ACE inhibitors, calcium channel blockers, hormones, NSAIDs, and other lipid-lowering therapy
Kaye, 2004, General Practice Research Database, UK	PC-CS	698/3267 (50–89 y)	No statin use Current statin use	1.0 (Ref) 0.9 (0.6–1.3)	Year of birth, sex, general practice, year of entry into the GPRD, and index date
Boudreau, 2004, western Washington State, US	PC-CS	975/1007 (65–79 y)	Nonuse Ever statin use	1.0 (Ref) 0.9 (0.7–1.2)	Age at reference date, reference year, county of residence, and use of antihypertensive medication
Kochhar, 2005, VISN 16 database, US	PC-CS	556/39865 (25–92 y)	Nonuse Ever statin use	1.0 (Ref) 0.49 (0.38–0.62)	Age, diabetes mellitus, smoking, alcohol consumption
Dumasia, 2006, southeastern Michigan, US	HC-CS	521/521 (35–101 y)	Nonuse Statin use for 4 + yrs	1.0 (Ref) 0.78 (0.47–1.31)	Age, race, BMI
Coogan, 2007, Philadelphia, New York, Baltimore, US	HC-CS	1185/3900 (40–79 y)	No statin use regular statin use	1.0 (Ref) 1.2 (0.8–1.8)	Age, interview year, study center, BMI, alcohol use, race, years of education, pack-years of smoking, NSAID use, use of conjugated estrogens or other female hormones, use of oral contraceptives, menopausal status, parity, age at menarche, family history of breast cancer and religion
Pocobelli, 2008, Wisconsin, Massachusetts, New Hampshire, US	PC-CS	3859/4761 (50y+)	No statin use Ever statin use for ≥0.5 y	1.0 (Ref)1.0 (0.8–1.2)	Age, state of residence, reference year, first degree family history of breast cancer, menopausal status/ageat menopause, parity/age at first birth, body mass index, recency of postmenopausal hormone use, education, and screening mammography history
Eaton, 2009, Fargo, ND, US	HC-CS	95/94 (55–81 yrs)	No statin use statin use	1.0 (Ref)1.3 (0.7–2.5)	Age, age at menopause, family history of breast cancer, parity
Woditschka, 2010, Northern California, US	PC-CS	HR negative: 3669/36690 (mean 59 y) HR positive:17195/171950 (mean 63)	HR negative: No statin use Statin use HR positive: No statin use Statin use	1.0 (Ref) 1.01 (0.94–1.09) 1.0 (Ref) 0.97 (0.94–1.004)	Birth year and duration of KPNC pharmacy coverage, oral contraceptive and hormone therapy use
Leung, 2015, NationalHealth Insurance Research Database, Taiwan	PC-CS	6463/18987	Nonuse Ever statin use	1.0 (Ref) 1.68 (1.52–1.85)	Age, comorbidities at cancer diagnosis, use of hormone replacement therapy
Vinogradova, 2011, 574 UK general practices, UK	NC-CS	15666/62938 (30 y+)	Nonuse Ever statin use	1.0 (Ref) 1.00 (0.93–1.08)	Townsend quintile, BMI, smoking status, myocardial infarction, coronary heart disease, diabetes, hypertension, stroke, rheumatoidarthritis, use of NSAIDs, Cox2-inhibitors, aspirin, family history of breast cancer, use of oral contraceptives, hormone-replace therapy
Blais, 2000, Canada	NC-CS	65/650	use of bile acid-binding resins use of statin	1.0 (Ref) 0.67 (0.33–1.38)	Age, previous neoplasm, year of cohort entry, use of other lipid-reducing agents, use of fibric acids, comorbidity score
Desai, 2013, Women's Health Initiative, US	CS	7430/154587 (50–79 y), mean 10.8 yrs	Nonuse Ever statin use	1.0 (Ref) 0.97 (0.87–1.08)	Age, BMI, ethnicity, smoking status, baseline hormone therapy use, baseline hormone therapy duration, family history of breast cancer, education, hysterectomy, mammogram last two years, age atFirst birth, parity, age at menarche, alcohol, percentage energy from fats, physical activity, and NSAID
Smeeth, 2008, The Health Improvement Network database, UK	CS	3204/729529 (40+ yrs), median 4.2 yrs	Nonuse Ever statin use	1.0 (Ref) 1.17 (0.95–1.43)	Age, propensity score, year of index date, first diagnosis of any of the following post-index date: diabetes, cerebrovascular disease, coronary heart disease, peripheral vascular disease, other atheroma, atrial fibrillation, heart failure, hyperlipidaemia, hypertension, other circulatory disease, cancer, dementia, first use of any of the following post-index date: aspirin, nitrates, fibrates, b-blockers, calcium channel blockers, potassium channel activators, diuretics, positive inotropes, anticoagulants, antihypertensives, or othercardiovascular drugs
Beck, 2003, Canada	CS	879/67472 (mean 61.3 yrs), mean 4.2 yrs	Nonuse Statin use	1.0 (Ref) 1.09 (0.93–1.28)	Age, sex
Jacobs, 2011, Cancer Prevention Study II Nutrition Cohort, US	CS	3070/73196 (NA), ∼8 yrs	Nonuse Statin use	1.0 (Ref) 1.05 (0.97–1.13)	NA
Haukka, 2009, Finland	CS	6046/NA (median 60 y), mean 8.8 yrs	Nonuse Statin use	1.0 (Ref) 1.01 (0.96–1.06)	Age, follow-up period
Cauley, 2003, Baltimore, Minneapolis, the Monongahela Valley, Portland, US	CS	244/7528 (mean 77 y), mean 6.8 yrs	Nonuse Statin use	1.0 (Ref) 0.30 (0.10–0.98)	Age, body weight, HRT, family history of breast cancer
Boudreau, 2007, western Washington State, US	CS	2707/92,788 (45–89 y), median 6.4 yrs	Nonuse Ever statin use	1.0 (Ref) 1.07 (0.88–1.29)	Age, use of hormone therapy, use of other lipid-lowering therapy, diabetes mellitus, BMI
Setoguchi, 2007, Pennsylvania, US	CS	300/31723 (65 y+), mean 2.9 yrs	Nonuse Ever statin use	1.0 (Ref) 0.99 (0.74–1.33)	Time, age, race, health service utilization, prevention-related activities including mammography and gynecological examination, diabetes, Arthritis, Inflammatory bowel diseases, Benign mammary dysplasia, Estrogen use, Estrogen-progesterone use, NSAID use, Use of gastroprotective drugs, obesity, Tobacco abuse diagnosis
Friis, 2005, Denmark	CS	3141/13508 (30–80 y), mean 3.3 yrs	Nonuse Ever statin use	1.0 (Ref) 1.02 (0.76–1.36)	Age, calendar period and use ofNSAIDs, hormone replacement therapy and cardiovascular drugs
Eliassen, 2005, NHS, US	CS	3177/75,828 (42–69 y), 6–12 yrs	Nonuse Current statin use	1.0 (Ref) 0.96 (0.83, 1.12)	Time, age, age at menarche, parity and age at first birth, height, body mass index, first-degree family history of breastcancer, benign breast disease, alcohol consumption, physical activity, and menopausal status, age at menopause, and use of postmenopausal hormones
Hsia, 2011, JUPITER, US	RCT	45/6205 (60+ y), 2.2 y	Control Statin group	1.0 (Ref) 0.94 (0.53–1.69)	NA
Nakamura, 2006, MEGA, Japan	RCT	25/5356 (mean 58 y), 4.6 y	Control Statin group	1.0 (Ref) 0.69 (0.31–1.53)	NA
HPS, 2005, UK	RCT	89/5082 (40–80 y), 5 y	Control Statin group	1.0 (Ref) 0.75 (0.49–1.13)	NA
Strandberg, 2004, SSSS, North Europe	RCT	12/827 (35–70 y), 10.4 y	Control Statin group	1.0 (Ref) 1.44 (0.46–4.52)	NA
Shepherd, 2002, PROSPER, North Europe	RCT	29/3000 (70–82 y), 3.2 y	Control Statin group	1.0 (Ref) 1.65 (0.78–3.48)	NA
Hague, 2003, LIPID, Oceania	RCT	17/1516 (31–75 y), 6.1 y	Control Statin group	1.0 (Ref) 1.13 (0.44–2.92)	NA
ALLHAT-LLT, 2002, North America	RCT	71/5051 (55+ y), 4.8 y	Control Statin group	1.0 (Ref) 0.93 (0.59–1.48)	NA
Sacks, 1996, CARE, North America	RCT	13/576 (mean 59 y), 4.8 y	Control Statin group	1.0 (Ref) 12.2 (1.59–93.0)	NA
Clearfield, 2001, AFCAPS, US	RCT	22/997 (55–73 y), 5.2 y	Control Statin group	1.0 (Ref) 1.44 (0.62–3.34)	NA

BMI: body mass index; CI: confidence interval; CS: cohort study; RCT: randomized clinical trial; HC-CS: hospital-based case-control study; NA: not available; NC-CS: nested case-control study; PC-CS: population-based case-control study; Ref: reference; RR: relative risk; HRT: Hormone replacement therapy; IDC: Invasive ductal carcinoma; ILC: Invasive lobular carcinoma; NSAID: Non-steroidal antiinflammatory drugs.

### Post-diagnosis statin use and breast cancer survival

**Figure 2:**
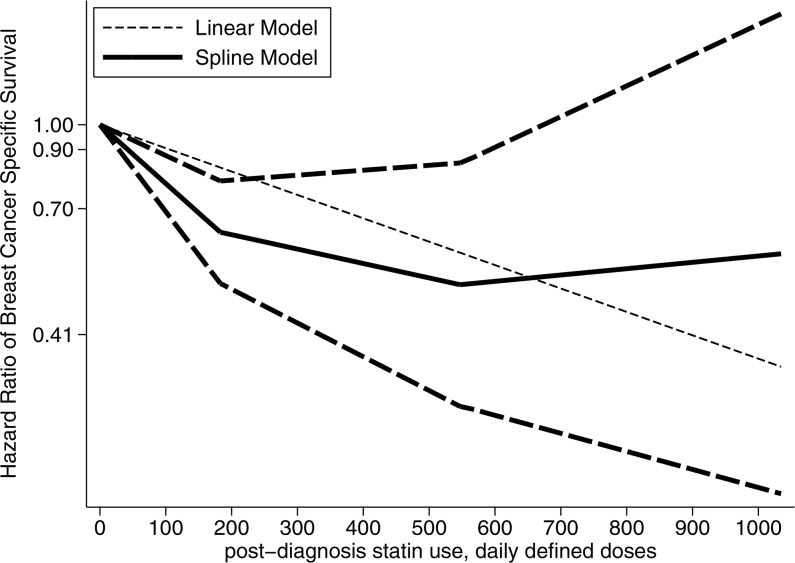
The dose-response relationship between post-diagnosis statin use and breast cancer specific survival

Four studies reported the association between post-diagnosis statin use and mortality of breast cancer patients [7–10]. These studies reported associations with overall survival (OS) and disease specific survival (DSS) respectively. For the study by Murtola et al [8], estimates according to subgroups of localized cases and metastatic cases were reported separately. We thus included both in the pooled analysis. Focusing on the association between post-diagnosis statin use and DSS of breast cancer patients, the pooled analysis of available studies revealed that there was a significantly negative association (HR = 0.65, 95% CI 0.43–0.98), with relatively considerable heterogeneity (I^2^ = 89.7%; Table 3). There was no significant publication bias as indicated by Egger's test (*p* for bias: 0.947) and Begg's test (*p* for bias: 1.000). The negative association was detected in studies focusing on general breast cancer patients, studies conducted in Europe and subgroup analysis according to follow up time (Table 3). There was a non-linear relationship between the duration of post-diagnosis statin use and DSS (p for likelihood ratio test: 0.0001) [7, 8]. The non-linear relationship was demonstrated in Figure 2.

**Table 3:** Summary risk estimates of the association between after-diagnosis statin use and breast cancer mortality

	No of reports	HR (95% CI)	I^2^ (%)	*P* for heterogeneity
**Overall mortality**	5	0.71 (0.48–1.07)	94.0	<0.001
**Subgroup analysis**				
**Breast cancer subtype**				
inflammatory breast cancer	1	1 (0.63–1.6)	–	–
more general breast cancer	4	0.67 (0.42–1.05)	95.3	<0.001
**Location**				
Europe	4	0.67 (0.42–1.05)	95.3	<0.001
America	1	1.00 (0.63–1.60)	–	–
**Follow up time (years)**				
<4.3 (median value)	3	**0.58 (0.35–0.95)**	87.0	<0.001
>4.3	2	0.94 (0.62–1.44)	83.9	0.013
**Disease specific mortality**	5	**0.65 (0.43–0.98)**	89.7	<0.001
**Subgroup analysis**				
**Breast cancer subtype**				
inflammatory breast cancer	1	0.95 (0.58–1.56)	–	–
more general breast cancer	4	**0.60 (0.38–0.95)**	91.7	<0.001
**Location**				
Europe	4	**0.60 (0.38–0.95)**	91.7	<0.001
America	1	0.95 (0.58–1.56)	–	–
**Follow up time (years)**				
<4.3 (median value)	3	**0.53 (0.31–0.91)**	84.8	0.001
>4.3	2	**0.77 (0.68–0.88)**	49.1	0.161

Regarding the association between post-diagnosis statin use and OS of breast cancer patients, there was not a statistically significant association (HR = 0.71, 95% CI 0.48–1.07; I^2^ = 94.0%; Table 3). There was no significant publication bias as indicated by Egger's test (*p* for bias: 0.992) and Begg's test (*p* for bias: 0.806). The subgroup analyses based on disease subtype, study location and follow up time also largely revealed null associations, although in the strata of shorter than 4.3 years of follow up time, the association reached statistical significance (HR = 0.58, 95% CI 0.35–0.95; Table 3).

### Pre-diagnosis statin use and breast cancer survival

Three studies reported the association between pre-diagnosis statin use and mortality of breast cancer patients [7, 8, 11], including both OS (2 studies) and DSS (3 studies). The study by Murtola et al [8] reported estimations for OS according to subgroups of localized cases and metastatic cases respectively. We thus included both in the pooled analysis of OS. Focusing on the association between pre-diagnosis statin use and OS of breast cancer patients, there was a negative association (HR = 0.68, 95% CI 0.54–0.84), with high heterogeneity (I^2^ = 75.7%; Table 4). There was no indication of publication bias according to Egger's test (*p* for bias: 0.541) and Begg's test (*p* for bias: 1.000). This inverse association persisted in subgroup analysis of follow up time.

**Table 4:** Summary risk estimates of the association between before-diagnosis statin use and breast cancer mortality

	No of reports	HR (95% CI)	I^2^ (%)	*P* for heterogeneity
**Overall mortality**	3	**0.68 (0.54–0.84)**	75.7	0.016
**Subgroup analysis**				
**Follow up time (years)**				
<4.5	2	0.60 (0.51–0.70)	0	0.505
>4.5	1	0.78 (0.70–0.86)	–	–
**Disease specific mortality**	3	**0. 72 (0.53–0.99)**	82.3	0.004
**Subgroup analysis**				
**Follow up time (years)**				
<4.5	1	0.54 (0.44–0.67)	–	–
>4.5	2	0.82 (0.72–0.93)	0	0.599

Similarly, we identified an inverse association between pre-diagnosis statin use and DSS of breast cancer patients, with a HR of 0.72 (95% CI 0.53–0.99). There was significant heterogeneity across studies (I^2^ = 82.3%; Table 4). The inverse association was detected in subgroups stratified by follow up time.

### Statin use and breast cancer risk

Among the 32 studies reporting the association between statin use and risk of breast cancer, two studies provided estimations according to different subgroups respectively [17, 28]. We included all these estimations in the overall pooled analysis because the overall estimation in these studies could not be inferred based on the available information. After summarizing results from all these studies, there was no significant association between statin use and breast cancer risk (RR = 1.02, 95% CI 0.95–1.09), with considerable heterogeneity (I^2^ = 80.8%; Table 5). No evident publication bias was detected based on the conducted Egger's test (*p* for bias: 0.730) and Begg's test (*p* for bias: 0.906). The null association persisted in almost all strata of subgroup analyses according to study design and location (Table 5).

**Table 5:** Summary risk estimates of the association between statin use and breast cancer risk

	No of reports	RR (95% CI)	I^2^ (%)	*P* for heterogeneity
**Overall estimation**	34	1.02 (0.95–1.09)	80.8	<0.001
**Subgroup analysis**				
**Location**				
Europe	9	1.01 (0.97–1.05)	0	0.600
America	22	0.99 (0.92–1.05)	63.8	<0.001
Asia	2	1.18 (0.50–2.77)	78.7	0.030
Oceania	1	1.13 (0.44–2.92)	–	–
**Study design**				
Prospective studies	12	1.02 (0.98–1.05)	0.0	0.462
Case-control studies	13	1.02 (0.88–1.18)	92.0	<0.001
RCTs	9	1.04 (0.78–1.39)	30.3	0.176

## DISCUSSION

We performed a comprehensive systematic review and meta-analysis to assess the relationship between statin use and mortality and risk of breast cancer. After summarizing all available evidence, it seemed that statin use was inversely associated with mortality of breast cancer patients, although the association between post-diagnosis statin use and OS did not reach statistical significance. An updated meta-analysis evaluating the relationship between statin use and breast cancer risk revealed a null association, which is consistent with previous analyses [2, 14, 51]. These findings demonstrated that the use of statin after diagnosis of breast cancer could potentially decrease mortality risk. Additionally, although statin use seemed not affect risk of breast cancer development, it might confer beneficial effect for decreasing the mortality of those individuals who develop breast cancer.

The detected beneficial effect of statin use on breast cancer mortality is plausible based on preclinical findings. Statins are shown to be able to inhibit growth in both breast cancer cell lines and *in-vitro* models [4, 52]. Research demonstrates that the anti-cancer effects may be induced by statins' effects on apoptosis, angiogenesis and tumor invasion [53–56]. For example, statins can regulate the mevalonate pathway, which is critical for the tumor promoting effects of p53 [57]. The dysregulation of this pathway is found to promote breast cancer tumor cell growth [58]. Besides, mevalonate stimulates tumor proliferation based on a mouse model [59]. Statins are also detected to potentially inhibit carcinogenesis through inhibiting isoprenoids [60]. Overall, these mechanistic understandings make the finding that statin use is inversely associated with breast cancer mortality to be more plausible. On the other hand, it is very interesting that statin use can decrease breast cancer mortality, and breast cancer recurrence [61], while has no effect on preventing incidence of breast cancer. Further research is needed to clarify why there are differentiated effects of statins on breast cancer development and prognosis.

The relationship between statin use and cancer has been extensively evaluated. Meta-analysis studies have suggested that use of statins is associated with reduced risks of liver cancer [62–64], ovarian cancer [65], colorectal cancer [66, 67], haematological malignancies [68], esophageal cancer [69, 70], gastric cancer [71, 72], and prostate cancer [73]. Furthermore, statins were demonstrated to be protective from breast cancer recurrence [61]. Our finding of the potential beneficial effect of statin use on breast cancer mortality is consistent with another newly published meta-analysis, in which statin use was suggested to be beneficial for overall cancer survival and cancer-specific survival, including breast cancer patients [74]. If the finding of the beneficial effect of statin use could be further replicated, it may be warranted to promote statin use to decrease public health burden from human cancers.

Our study has several strengths. To the best of our knowledge, up to date this is the most comprehensive meta-analysis study evaluating the relationship between statin use and breast cancer, including assessing the associations between post-diagnosis and pre-diagnosis stains use and breast cancer mortality, as well as the dose-response relationship. Compared with previous meta-analyses evaluating the relationship between statin use and breast cancer risk [2, 14], our study incorporates evidence from more recent studies and is more comprehensive. Compared with another meta-analysis assessing the effect of statin use on cancer mortality [74], our analysis includes evidence from more relevant studies for breast cancer, as well as evaluates the dose-response relationship for the association. Our study adds new knowledge for the relationship between statin use and cancers, and provides further evidence for supporting the use of statins for decreasing health burden from cancers, if the findings of the current study could be replicated by additional studies.

Several potential limitations must be acknowledged for the appropriate interpretation of our findings. First, we did not have access to the individualized primary data from the included studies, and the risk estimates used in the pooled analyses might not be fully adjusted. Although almost all included studies provided adjusted estimates considering important confounders, residual confounding may be an issue for biasing the results. Second, our main finding of the potential beneficial effect of statin use on breast cancer survival may be biased somehow due to that evidence was from observational studies, which are known to confer incoherent shortcomings. For the included studies evaluating statin use with mortality, there are relatively large differences in the definitions of statin use [8, 10], which may bias the results. Further large scale well designed studies are warranted to replicate our findings. Third, we detected considerable heterogeneity in the pooled analyses, which were not eliminated in the subgroup analyses. Additionally, the findings of the association between statin use and breast cancer mortality were based on limited studies, although these are the currently available evidence for evaluating this relationship. In spite that we detected a non-linear dose-response relationship for the association between statin use and breast cancer mortality, data were merely based on two available studies. Further research will be critical to more accurately clarify and resolve these issues.

In conclusion, based on a summarization of all available evidence, statin use was inversely associated with breast cancer mortality, although statin use was not associated with breast cancer risk. If replicated in further large-scale well designed studies, our findings may have implication for supporting the use of statins for decreasing health burden from breast cancer.

## MATERIALS AND METHODS

### Data sources and search strategies

A comprehensive search of PubMed (MEDLINE), Web of Science, and Embase databases was conducted from each database's inception to January 2015 for studies published in English Controlled vocabulary and keywords were used to search for eligible studies. The detailed search strategy is described in the Supplementary Material. We also screened references of relevant review articles to identify other potential studies. The literature search was updated at August 2015.

### Study selection

Studies were eligible if they (i) were case–control studies, prospective studies, or RCTs; (ii) evaluated the association between statin use and breast cancer risk or survival; (iii) presented relative risk (RR), odds ratio (OR), hazard ratio (HR) estimates with 95% confidence intervals (CI) or necessary data for determination. There was no restriction for sample size and follow-up duration. If there were several publications from the same study, we included the study with the most cases and relevant information, like our previous studies [75–78].

### Data extraction and quality assessment

A pair of investigators independently carried out the abstract screening, full text screening, and data extraction (Chao Tu and Lang Wu). Disagreements were resolved by discussion, with input from other investigators. Data extracted from each study included: the first author's last name, year of publication, study location, study design, characteristics of study population (sample size, age, length of follow-up), and effect sizes of the associations. If multiple estimates of the association for the same outcome were reported, we used the estimate that was adjusted for the most appropriate covariates, like previous studies [79–81]. In situations when only unadjusted estimates were given, we used the crude estimate.

The quality assessment of included studies were performed according to the Newcastle-Ottawa Quality Assessment Scale (NOS) for observational studies [82, 83], and a revised form of Cochrane Collaboration's tool for assessing risk of bias in randomized trials [84, 85]. For NOS population and sampling methods, exposure and outcome, as well as statistical matching/adjustments were considered. Studies with assigned scores of at least 7 were categorized as high quality studies. For RCTs the adequacy of randomization, allocation concealment, as well as blinding were evaluated. Studies were categorized as with low risk of bias only if all of these items were adequately described as with low risk.

### Statistical methods

Due to the relative rarity of breast cancer in the general population, ORs and HRs were deemed equivalent to RRs. We used RRs to represent measures of studies evaluating associations with breast cancer risk. For studies evaluating breast cancer survival, HRs were used since all involved studies used HRs as the estimation. We used the I^2^ to evaluate the heterogeneity across studies, in which a I^2^ > 50% suggests high heterogeneity [86]. We pooled the log transformed RR or HR using the fixed-effects model [87] if there was no considerable heterogeneity. If there was substantial heterogeneity, we used the random-effects model [88]. With regards to the relationship between statin use and breast cancer survival, since for two included studies [7, 8], the associations of both post-diagnosis statin use and pre-diagnosis statin use were provided separately, we conducted analyses for pre-diagnosis statin use and post-diagnosis statin use respectively. Subgroup analyses were also conducted based on disease subgroup, study design (case-control vs. prospective studies vs. RCTs) and study geographic location (Europe, America or Asia) for studies evaluating disease risk, and disease subgroup, study geographic location and follow up time for studies evaluating disease mortality.

For the dose-response analysis, we explored potential non-linear relationship between the duration of statin use and breast cancer mortality, using principles as previously published [89]. For studies reporting statin use by categories, we used the midpoint of each category to represent the exposure. If the highest category did not provide the upper bound, we assumed the open ended interval's width to be as same as the adjacent interval's width. We examined a potential nonlinear dose-response relationship between statin use and breast cancer survival with fractional polynomial models, using restricted cubic splines with 3 knots at fixed percentiles (10%, 50% and 90%) of the distribution [90, 91]. We also conducted likelihood ratio tests to determine whether nonlinear or linear relationship was suggested.

Publication bias was evaluated via Egger's test [92] and Begg's test [93]. A *P*-value of 0.05 was used to determine whether there was significant publication bias. All statistical analyses were performed with Stata (version 13; StataCorp, College Station, TX).

## SUPPLEMENTARY FIGURES AND TABLES



## Abbreviations

(OR): Odds ratio
(RR): relative risk
(HR): hazard ratio
(CI): confidence intervals
(RCT): randomized clinical trials
(OS): overall survival
(DSS): disease specific survival
