# Supplementary material for: Statin use and breast cancer survival and risk: a systematic review and meta-analysis
Source: Oncotarget. 2015 Oct 12;6(40):42988–3004. doi: 10.18632/oncotarget.5557 (PMC4767486; doi:10.18632/oncotarget.5557)
Supplement: Supplementary file 1 [file oncotarget-06-42988-s001.pdf]

SUPPLEMENTARY DATA

Literature search strategy

We used the following search keywords: ((malignancies[Title/Abstract] OR malignancy[Title/Abstract]) OR (((((((neoplasm[Title/Abstract] OR neoplasms[Title/Abstract]) OR cancer[Title/Abstract]) OR cancers[Title/Abstract]) OR adenoma[Title/Abstract]) OR adenomas[Title/Abstract]) OR carcinoma[Title/Abstract]) OR carcinomas[Title/Abstract])) AND ((“hydroxymethylglutaryl-coa reductase inhibitors”[Pharmacological Action] OR “hydroxymethylglutaryl-coa reductase inhibitors”[MeSH Terms] OR (“hydroxymethylglutaryl-coa”[All Fields] AND “reductase”[All Fields] AND “inhibitors”[All Fields]) OR “hydroxymethylglutaryl-coa reductase inhibitors”[All Fields] OR “statin”[All Fields]) OR (“hydroxymethylglutaryl-coa reductase inhibitors”[Pharmacological Action] OR “hydroxymethylglutaryl-coa reductase inhibitors”[MeSH Terms] OR (“hydroxymethylglutaryl-coa”[All Fields] AND “reductase”[All Fields] AND “inhibitors”[All Fields]) OR “hydroxymethylglutaryl-coa reductase inhibitors”[All Fields] OR “statins”[All Fields])).

Supplementary Table S1: Quality Assessment of Included Prospective Cohort Studies for Breast Cancer Mortality

Study	Exposed cohort represents average in community	Selection of the non-exposed cohort from same community	Ascertain exposure through records or structured interviews	Demonstrate that outcome not present at study start	Exposed and non-exposed matched and/or adjusted by factors	Ascertain outcome via independent blind assessment or record linkage	Follow-up long enough for outcome to occur	Loss to follow-up <20%	Overall Score
Brewer, 2013	1	1	1	1	2	1	1	1	9
Cardwell, 2014	1	1	1	1	2	1	1	1	9
Murtola, 2014	1	1	1	1	2	1	1	0	8
Nickels, 2013	1	1	1	1	2	1	1	1	9
Desai, 2015	1	1	1	1	2	1	1	1	9

1 means study adequately fulfilled a quality criterion, 0 means it did not. Quality scale does not imply that items are of equal relevant importance

Supplementary Table S2: Quality Assessment of Included Prospective Studies for Breast Cancer Risk

Study	Exposed cohort represents average in community	Selection of the non-exposed cohort from same community	Ascertain exposure through records or structured interviews	Demonstrate that outcome not present at study start	Exposed and non-exposed matched and/or adjusted by factors	Ascertain outcome via independent blind assessment or record linkage	Follow-up long enough for outcome to occur	Loss to follow-up <20%	Overall Score
Desai, 2013	1	1	1	1	2	1	1	0	8
Smeeth, 2008	1	1	1	1	2	1	1	0	8
Beck, 2003	1	1	1	1	2	1	1	0	8
Jacobs, 2011	1	1	0	1	0	0	1	1	5
Haukka, 2009	1	1	1	1	2	1	1	0	8
Cauley, 2003	1	1	1	1	2	1	1	0	8
Boudreau, 2007	1	1	1	1	2	1	1	0	8
Setoguchi, 2007	1	1	1	0	2	1	1	0	7
Friis, 2005	1	1	1	1	2	1	1	0	8
Eliassen, 2005	0	1	0	1	2	1	1	1	7
Vinogradova, 2011	1	1	1	1	2	0	1	1	8
Blais, 2000	1	1	1	0	2	1	1	1	8

1 means study adequately fulfilled a quality criterion, 0 means it did not. Quality scale does not imply that items are of equal relevant importance

Supplementary Table S3: Quality Assessment of Included Case-Control Studies for Breast Cancer Risk

Study	Case defined with independent validation	Represen-tativeness of the cases	Selection of controls from community	Statement that controls have no history of outcome	Cases and controls matched and/or adjusted by factors	Ascertain exposure by blinded structured interview	Same method of ascertainment for cases and controls	Same response rate for both groups	Overall score
McDougall, 2013	1	1	1	0	2	1	0	1	7
Graaf, 2004	0	1	0	1	2	1	1	1	7
Kaye, 2004	1	1	1	0	2	1	1	1	8
Boudreau, 2004	0	1	1	0	2	1	1	1	7
Kochhar, 2005	0	0	1	0	2	1	1	1	6
Dumasia, 2006	0	1	1	0	2	0	1	1	6
Coogan, 2007	1	1	0	1	2	1	1	1	8
Pocobelli, 2008	0	1	1	1	2	1	1	1	8
Eaton, 2009	1	1	0	0	2	1	1	1	7
Woditschka, 2010	0	1	1	0	2	1	1	1	7
Leung, 2015	0	1	1	1	2	1	1	1	8

1 means study adequately fulfilled a quality criterion, 0 means it did not. Quality scale does not imply that items are of equal relevant importance

Supplementary Table S4: Quality Assessment of Included Randomized Clinical Trials for Breast Cancer Risk

Study	Adequacy of randomization	Allocation concealment	Blinding
Hsia, 2011	adequate	not adequately described	adequate
Nakamura, 2006	adequate	not adequately described	inadequate
HPS, 2005	adequate	adequate	adequate
Strandberg, 2004	not adequately described	not adequately described	adequate
Shepherd, 2002	adequate	adequate	adequate
Hague, 2003	adequate	not adequately described	adequate
ALLHAT-LLT, 2002	adequate	adequate	inadequate
Sacks, 1996	adequate	not adequately described	adequate
Clearfield, 2001	not adequately described	not adequately described	adequate
